# Assessment of Autonomic Nervous System Function in Patients with Aortic Stenosis and Diabetes Mellitus

**DOI:** 10.3390/diagnostics16060871

**Published:** 2026-03-15

**Authors:** Mihajlo Farkić, Nikola Marković, Valentina Balint, Maša Petrović, Milovan Bojić, Branislav Milovanović

**Affiliations:** 1 Institute for Cardiovascular Diseases “Dedinje”, 11000 Belgrade, Serbia; 2 Faculty of Medicine, University of Banja Luka, 78000 Banja Luka, Bosnia and Herzegovina; 3 School of Medicine, University of Belgrade, 11000 Belgrade, Serbia

**Keywords:** aortic stenosis, diabetes mellitus, autonomic nervous system, heart rate variability, electrocardiography, TAVR

## Abstract

**Background/Objectives:** Aortic stenosis is associated with autonomic nervous system (ANS) imbalance, while diabetes mellitus is a major contributor to cardiac autonomic neuropathy. Their coexistence may result in more pronounced autonomic dysfunction not fully captured by conventional assessment. This study aimed to compare ANS function in patients with severe aortic stenosis undergoing transcatheter aortic valve replacement (TAVR), according to diabetes status. **Methods:** This cross-sectional study included 74 patients with severe aortic stenosis referred for TAVR, including 21 patients with diabetes mellitus. Autonomic function was evaluated using non-invasive ECG-based analysis, incorporating short-term and 24 h Holter-derived heart rate variability (HRV), nonlinear Poincaré plot indices, and deceleration and acceleration capacity. Ambulatory blood pressure monitoring and standard clinical and echocardiographic assessment were performed. **Results:** Patients with diabetes mellitus demonstrated significantly lower long-term HRV parameters and reduced nonlinear Poincaré plot indices compared with non-diabetic patients, indicating altered autonomic modulation. Short-term HRV showed similar trends without statistical significance. Echocardiographic severity of aortic stenosis and left ventricular systolic function were comparable between groups. **Conclusions:** Autonomic dysfunction appears to be more pronounced in patients with severe aortic stenosis and diabetes mellitus, predominantly affecting parasympathetic modulation. ECG-derived autonomic parameters may offer complementary insight into ANS involvement in this population and warrant further investigation.

## 1. Introduction

Aortic stenosis (AS) is the most prevalent valvular heart disease in developed countries and represents a major cause of cardiovascular morbidity and mortality, particularly among older adults. Its prevalence increases markedly with age, affecting approximately 2–4% of individuals older than 65 years, with degenerative calcification of the aortic valve constituting the predominant etiology [1,2]. Degenerative AS is now recognized as an active and tightly regulated pathological process rather than a passive consequence of aging. Valve calcification represents the final common pathway of lipid infiltration, chronic inflammation, osteogenic signaling, and activation of valve interstitial cells [3,4]. Progressive valvular obstruction leads to chronic left ventricular pressure overload, promoting concentric hypertrophy, impaired ventricular compliance, and reduced cardiac reserve, ultimately predisposing patients to heart failure and adverse clinical outcomes [5,6].

Within this pathophysiological framework, diabetes mellitus (DM) has emerged as an important modifier of AS development and progression. Chronic hyperglycemia, insulin resistance, and systemic inflammation accelerate valvular mineralization, enhance left ventricular hypertrophy, and contribute to myocardial dysfunction [7,8,9]. Population-based studies indicate that individuals with diabetes have up to a 70% higher risk of aortic valve calcification and incident AS compared with non-diabetic individuals, even at subdiabetic glucose levels [10]. Among patients with established AS, the presence of DM is associated with impaired myocardial performance, increased cardiovascular mortality, and a higher incidence of adverse events, including heart failure and sudden cardiac death [11].

In addition to structural and hemodynamic alterations, AS is characterized by significant disturbances in autonomic nervous system (ANS) regulation secondary to chronic pressure overload. Progressive valvular obstruction is associated with heightened sympathetic activation and reduced parasympathetic tone, resulting in resting tachycardia, diminished heart rate variability (HRV), and impaired cardiovascular reflex responses [12,13]. Emerging evidence suggests that autonomic dysfunction in AS contributes to arrhythmogenesis, exercise intolerance, and adverse clinical outcomes, some of which may partially improve following valve replacement [14,15].

DM is also a well-established cause of cardiac autonomic neuropathy (CAN) 16]. CAN represents a common yet frequently under-recognized microvascular complication of diabetes, with reported prevalence reaching up to 70% in long-standing disease [16]. Early stages are typically characterized by predominant parasympathetic impairment, whereas sympathetic dysfunction tends to develop later in the disease course [16]. Once established, CAN is associated with a substantially worse prognosis, including a five-year mortality rate ranging from 25% to 50% [16]. Importantly, CAN contributes to the development and progression of several cardiovascular conditions prevalent in diabetes—such as heart failure, atrial fibrillation, and coronary artery disease–particularly in the context of silent or unrecognized myocardial ischemia [17,18,19].

Given the overlapping pathophysiological mechanisms of AS and DM, particularly with respect to autonomic dysfunction, a synergistic effect on cardiovascular regulation may be anticipated. However, data directly comparing autonomic function in patients with AS according to diabetic status remain limited.

The aim of this study was to evaluate and compare ANS function, primarily using short- and long-term ECG analysis, with derived parameters of interest, as well as ambulatory blood pressure monitoring in patients with severe AS undergoing Heart Team–guided transcatheter aortic valve replacement (TAVR), stratified by the presence or absence of DM.

## 2. Materials and Methods

### 2.1. Study Protocol

This cross-sectional comparative study included 74 adult patients (≥18 years) with severe aortic stenosis (AS) and a Heart Team–established indication for transcatheter aortic valve replacement (TAVR). All participants were hospitalized for the planned TAVR procedure at the Institute for Cardiovascular Diseases “Dedinje” between January 2025 and November 2025.

During hospitalization, autonomic nervous system (ANS) assessment was performed in the Neurocardiological Laboratory according to the predefined study protocol. Among the 74 participants, 21 patients (28.4%) had a confirmed diagnosis of diabetes mellitus (DM) and were allocated to the DM group, while the remaining 53 patients comprised the non-DM group.

Inclusion criteria were severe AS and a TAVR indication established in accordance with current clinical guidelines [20]. The diagnosis of DM was confirmed by an endocrinologist based on established diagnostic criteria.

Exclusion criteria included absence of sinus rhythm (permanent pacemaker, atrial fibrillation, atrial flutter, atrial tachycardia), acute hemodynamic instability (e.g., decompensated heart failure or significant dyspnea at rest), primary autonomic neuropathies (e.g., multiple system atrophy, Parkinson’s disease, multiple sclerosis), and severe respiratory or renal failure. Patients with incomplete, technically inadequate, or unreliable recordings were also excluded.

The study was conducted in accordance with the principles of the Declaration of Helsinki and approved by the Scientific Ethics Committee of the Institute for Cardiovascular Diseases “Dedinje” (approval number 339; date of approval 26 January 2026).

Collected data included demographic characteristics (age, sex, body mass index), detailed comorbidity profiles, cardiovascular and non-cardiovascular conditions, current cardiovascular pharmacotherapy, and routine transthoracic echocardiographic findings obtained within six months prior to the intervention.

During hospitalization, one day prior to TAVR, patients underwent standardized 5 min resting ECG recording for short-term heart rate variability (HRV) analysis, followed by 24 h Holter ECG monitoring and 24 h ambulatory blood pressure monitoring (ABPM).

### 2.2. 5 min ECG Recording

Five-minute ECG recordings were made in the supine resting position, using a standard 12-lead electrocardiogram. The following parameters were observed: Heart rate (HR), average duration of following waves and intervals: P, QRS, T, QT and QTc, alongside with parameters of Heart Rate Variability: SDNN (Standard deviation of normal RR intervals), RMSSD (Root Mean Square of Successive Differences), PNN50 (Percentage of successive NN intervals that differ by more than 50 milliseconds), TI (Triangular index), TP (Total power), VLF (Very Low Frequency), LF (Low Frequency) and HF (High frequency) and LF/HF.

### 2.3. 24 h Holter ECG Monitoring

Twenty-four-hour ECG monitoring was conducted using a three-lead recording system (CardioScan, DM Software Inc., Stateline, NV, USA) All recordings were carefully inspected by an experienced clinician, with manual verification and correction of beat labeling prior to further processing. After artifact removal and rhythm annotation adjustments, HRV analysis was performed using the integrated software. HRV parameters obtained from 24 h recordings included the same indices as those derived from the short-term (5 min) ECG assessment, with the addition of the ultra-low-frequency (ULF) component. Nonlinear HRV analysis was performed using Poincaré-plot–based measures: VLI (Vector Length Index), VAI (Vector Angle Index), L (Poincare length), D (Poincare dispersion), SD1, SD2, SD1/SD2, SD2/SD1, LA (Poincare length) and SA (Poincare width). Using dedicated commercial software, deceleration capacity (DC) and acceleration capacity (AC) were also computed. The average number of premature ventricular contractions (PVCs) and premature atrial contractions (PACs) during 24 h was recorded. Heart rate turbulence (HRT) parameters—turbulence onset (TO) and turbulence slope (TS)—were calculated only in patients who met the required technical criteria (primarily the presence of a sufficient number of analyzable PVCs) and categorized as normal/abnormal, according to established criteria [21]. Consequently, TO and TS were successfully derived in 12 patients in the DM group and in 31 patients in the non-DM group.

### 2.4. Ambulatory Blood Pressure Monitoring

Ambulatory blood pressure monitoring was performed using a validated oscillometric device, with measurements obtained every 30 min during daytime and every 60 min during nighttime over a 24 h period. Mean daytime (8:00–22:00) and nighttime (22:00–8:00) systolic and diastolic blood pressure values, together with their standard deviations, were calculated. Dipping status was determined according to the relative nocturnal decline in systolic blood pressure: (1) dipper (10–20% nocturnal reduction), (2) non-dipper (0–10% reduction), (3) extreme dipper (>20% reduction), and (4) reverse dipper (increase in nocturnal values). In addition, the percentage of readings above predefined diagnostic thresholds was calculated. Thresholds were set at ≥140 mmHg (daytime) and ≥125 mmHg (nighttime) for systolic blood pressure, and ≥90 mmHg (daytime) and ≥80 mmHg (nighttime) for diastolic blood pressure.

### 2.5. Statistical Analysis

Data are presented as mean ± standard deviation (SD), median (Mdn) with interquartile range (IQR, 25–75%), which depends on the type of distribution for continuous variables or counts (percentage), depending on data type. Normality of continuous variables was assessed using the Smirnov test and by visual inspection of histograms and Q–Q plots. Group comparisons were performed using parametric tests (independent Student’s *t*-test) or nonparametric tests (Chi Square test, Fisher’s exact test, and Mann–Whitney U test) for categorical or non-normally distributed continuous data. Statistical analyses were conducted using SPSS version 26.0. A two-sided *p* value < 0.05 was considered statistically significant.

## 3. Results

Baseline demographic and clinical characteristics of the study population are presented in Table 1.

Patients with diabetes mellitus (DM) had significantly higher body mass index (BMI) compared with patients without DM (28.5 ± 5.1 vs. 25.5 ± 4.2 kg/m^2^, *p* = 0.010). No other statistically significant differences were observed between the groups.

The majority of patients were female and had a high prevalence of hypertension, hyperlipoproteinemia, and prior coronary artery disease. Approximately 20% of patients in both groups had atrioventricular (AV) block. Functional status according to NYHA classification and the prevalence of angina or prior syncope did not differ significantly between groups.

Table 2 shows the medications used in the study population. Patients with DM were more frequently treated with dihydropyridine calcium channel antagonists (DHP-CCAs) (61.9% vs. 34.6%, *p* = 0.033). No other statistically significant differences in pharmacotherapy were observed.

Although sodium–glucose cotransporter-2 inhibitors (SGLT2i) were more frequently prescribed in the non-DM group (32.7% vs. 28.6%), this difference was not statistically significant (*p* = 0.732).

Echocardiographic characteristics are shown in Table 3. No statistically significant differences were observed between the DM and non-DM groups. Mean left ventricular ejection fraction (LVEF) was preserved in both groups (51.2 ± 8.6% vs. 50.1 ± 9.6%, *p* = 0.652). Markers of AS severity were comparable, with mean aortic valve area (AVA) approximately 0.7 cm^2^ and mean transaortic pressure gradient around 50–57 mmHg in both groups. Left ventricular dimensions, interventricular septal thickness, right ventricular systolic pressure, and the prevalence of wall motion abnormalities were similar between groups.

Table 4 shows ECG and short-term Heart Rate Variability parameters. The P-wave duration was significantly shorter in the DM group compared with the non-DM group (94.8 ± 18 ms vs. 107.8 ± 20 ms, *p* = 0.012). No other ECG interval differences reached statistical significance.

Although most short-term HRV parameters (SDNN, RMSSD, pNN50, LF, HF, and total power) were numerically lower in the DM group, none achieved statistical significance. The pNN50 parameter approached statistical significance (*p* = 0.058).

Twenty-four–hour Holter ECG and long-term HRV parameters are presented in Table 5. SDNN and SDANN, as well as PNN50 and TI were statistically lower in the DM group. Additionally, HF was also found to be statistically lower in the DM group when compared to Non-DM group. Although other HRV parameters, as well as DC and AC were lower in DM group, they did not reach levels of statistical significance.

Poincaré plot–derived parameters are shown in Table 6. Nearly all parameters of Poincaré plot–derived parameters, with the exception of SD2/SD1, were lower in the DM group. Additionally, nearly all parameters, with the exception of SD1 reached levels of statistical significance.

Ambulatory blood pressure monitoring parameters are presented in Table 7. Mean systolic and diastolic blood pressure values (24 h, daytime, and nighttime averages) were comparable between groups and did not differ significantly. SD of systolic blood pressure during day was lower in DM group. Most patients in both groups exhibited a non-dipping systolic blood pressure pattern, with no statistically significant difference in dipping status.

The proportion of daytime systolic blood pressure readings above the predefined threshold was significantly higher in the non-DM group (49% vs. 23.8%, *p* = 0.049). No other differences in threshold exceedance were observed.

## 4. Discussion

In this cross-sectional cohort of patients with severe aortic stenosis (AS) referred for Heart Team–guided TAVR, diabetes mellitus (DM) was associated with a distinct pattern of autonomic dysregulation. Autonomic impairment was most consistently detected using long-term heart rate variability (HRV) indices and nonlinear measures derived from Poincaré plot analysis. Overall, the observed HRV profile in the DM group—particularly the reduction in vagally mediated indices (pNN50 and HF)—supports a predominant attenuation of parasympathetic modulation in this population.

Baseline demographic and clinical characteristics were broadly comparable between groups, with the exception of a higher BMI in the DM group (Table 1). This finding is consistent with the metabolic phenotype commonly accompanying type 2 DM and may contribute to autonomic imbalance through adiposity-related inflammation, neurohumoral activation, and altered cardiometabolic signaling [22]. Importantly, the absence of between-group differences in age, symptom burden, coronary artery disease prevalence, and conduction abnormalities suggests that the autonomic differences observed in patients with DM are unlikely to be explained solely by more advanced structural heart disease or electrical substrate (Table 5 and Table 6). Rather, these results are compatible with diabetes-specific metabolic and neurohormonal mechanisms contributing to autonomic impairment.

Pharmacotherapy was largely similar across groups, reflecting consistent application of guideline-directed medical therapy (Table 2). Although calcium channel antagonists were more frequently used in the DM group, the overall medication profiles were comparable, reducing the likelihood that the principal HRV differences were primarily medication-driven. Prior studies have shown that autonomic dysfunction in DM persists even after adjustment for cardiovascular therapies, supporting the concept of intrinsic diabetic CAN [23].

Echocardiographic characteristics were also comparable between groups, including left ventricular systolic function, chamber dimensions, and indices of AS severity (Table 3). This is clinically relevant, as it suggests that diabetes-related autonomic impairment may occur without overt differences in conventional echocardiographic parameters. Previous work has demonstrated that DM may worsen myocardial stiffness and diastolic function even when ejection fraction and valvular hemodynamics are similar [24]. Our findings extend this concept by indicating that autonomic dysregulation may represent an additional—potentially early and clinically meaningful—manifestation of diabetes-related myocardial involvement in severe AS.

An interesting finding in short-term ECG analysis was a significantly shorter P-wave duration in the DM group (Table 4). P-wave duration reflects atrial depolarization and intra-atrial conduction [25], and prolonged values have been associated with atrial remodeling and a higher risk of atrial fibrillation [26,27]. While studies in AS have reported longer P-wave duration [28,29], shorter P-wave duration has been described in patients with diabetes mellitus [30]. Atrial conduction is also influenced by autonomic nervous system activity, with sympathetic stimulation shortening and parasympathetic activity prolonging atrial conduction time [31,32]. Therefore, the shorter P-wave duration observed in the DM group may reflect both autonomic influences and underlying structural atrial remodeling. However, as a detailed echocardiographic assessment of the left atrium (e.g., LAVI or left atrial strain) was not performed, further studies integrating advanced echocardiographic parameters are needed to clarify the underlying mechanisms.

Since none of the standard ECG parameters significantly differed between the two groups, it may be useful to consider emerging non-invasive electrocardiographic indices that provide additional insight into cardiac electrophysiology. One such parameter is the cardio-electrophysiological balance index (iCEB), calculated as the ratio between the QT interval and QRS duration (QT/QRS) [33]. This simple metric reflects the balance between ventricular depolarization and repolarization and has been proposed as a potential marker of arrhythmogenic risk in various cardiovascular conditions. Therefore, evaluating such novel electrocardiographic markers may offer additional perspectives beyond conventional ECG parameters.

Long-term HRV analysis demonstrated significantly lower time-domain indices in patients with DM, including SDNN, SDANN, triangular index, and pNN50 (Table 5). SDNN, as a marker reflecting overall autonomic nervous system activity, has been consistently associated with increased cardiovascular morbidity and mortality, with lower values indicating impaired autonomic regulation [34,35,36]. In addition, both patients with DM and those with AS have been shown to exhibit lower SDNN values compared with healthy individuals [37,38]. Beyond SDNN, SDANN—which reflects circadian autonomic modulation—and the triangular index, a geometric measure of overall autonomic variability, were also reduced in patients with DM in the present study. These findings are consistent with previous reports demonstrating lower values of these parameters in individuals with DM and AS compared with healthy populations [36,37,38]. Importantly, pNN50—predominantly reflective of parasympathetic modulation—was significantly lower in the DM group, supporting vagal withdrawal as a central component of the observed autonomic profile [34,35].

Frequency-domain parameters further supported this interpretation. The DM group exhibited significantly lower ULF and HF power (Table 5). ULF reflects very slow oscillations related to circadian rhythms and long-term autonomic modulation and has been associated with increased mortality in several cohorts, particularly following acute coronary syndromes [39,40,41]. On the other hand, HF, the so-called respiratory band, represents a well-established marker of parasympathetic activity and has been shown to correlate more consistently with vagal modulation than pNN50 in certain settings [34]. Taken together, the concurrent reduction in time-domain parameters (SDNN, SDANN, TI, pNN50) and frequency-domain measures (ULF, HF) suggests the presence of impaired autonomic regulation in patients with DM, predominantly involving parasympathetic withdrawal, which may contribute to an overall less favorable cardiovascular risk profile compared with patients without DM.

As shown in Table 6, the majority of Poincaré plot parameters were statistically reduced in patients with DM, except for SD1, SD1/SD2, and SD2/SD1. Poincaré plot analysis is a visual and quantitative approach that characterizes beat-to-beat heart rate dynamics through the geometric shape of the plot, thereby providing insight into autonomic regulation [42]. Its application is supported by the fact that heart rate control represents a nonlinear process that cannot be fully captured by conventional linear measures, while isolated outliers such as premature atrial or ventricular contractions (PACs, PVCs) have a weaker influence on the Poincaré plot compared with other HRV parameters [42,43]. The most extensively studied Poincaré plot parameters are SD1, which reflects rapid beat-to-beat variability in RR intervals and predominantly represents parasympathetic activity, and SD2, which corresponds to long-term RR interval variability and is influenced by both sympathetic and parasympathetic modulation [44]. Guzik previously demonstrated reduced values of both SD1 and SD2 in patients with diabetes mellitus compared with healthy controls, while Khandoker et al. reported similarly reduced SD1 and SD2 values in diabetic patients with confirmed cardiac autonomic neuropathy compared with those without neuropathy [45,46]. Other parameters derived from the Poincaré plot have been less extensively studied, with most available evidence focusing on the variability and asymmetry indices, VLI and VAI. In patients with heart failure, Wang et al. reported reduced values of both VLI and VAI, indicating that decreased parasympathetic activity combined with increased sympathetic drive leads to shortening of the mean RR interval and a parallel reduction in both linear and nonlinear HRV indices, including SDNN, LFn, VAI, and VLI [47]. Overall, these Poincaré plot findings are in line with long-term heart rate variability results from time- and frequency-domain analyses and suggest impaired autonomic regulation in patients with DM, mainly due to reduced parasympathetic activity.

One important aspect is that in DM, as a consequence of the underlying pathophysiology of the disease, damage first occurs in the longest nerve, which in this case is the vagal nerve, the main nerve of the parasympathetic nervous system [16]. CAN in DM is thought to develop as a result of advanced glycation end products, increased oxidative stress, alterations in metabolic pathways, as well as ischemia associated with microvascular angiopathy [16]. Apart from that, diabetes-related myocardial stiffness, concentric remodeling, and diastolic dysfunction may impair ventricular filling and arterial–cardiac coupling, thereby increasing sympathetic drive and limiting parasympathetic modulation [24,48]. DM may also exacerbate autonomic dysregulation indirectly by accelerating aortic valve calcification and altering ventricular loading conditions. Degenerative AS is increasingly recognized as an active inflammatory and osteogenic process, and DM may promote calcification through chronic hyperglycemia, insulin resistance, and systemic low-grade inflammation [7,9]. Increased valvular stiffness and reduced arterial compliance may blunt baroreflex responsiveness, contributing to impaired cardiovascular reflex control in diabetic patients with severe AS, in addition to the primary effects of diabetic autonomic neuropathy.

In ABPM analysis, mean systolic and diastolic blood pressure values (24 h, daytime, and nighttime) were similar between groups (Table 7). However, indices of blood pressure variability differed: the non-DM group exhibited higher daytime systolic BP variability (SD of daytime SBP) and higher nighttime diastolic BP variability (SD of nighttime DBP), and a greater proportion of daytime SBP readings exceeding the predefined threshold. Increased BP variability has been associated with hypertension and adverse cardiovascular outcomes, including mortality, in population studies [49,50]. Given the more pronounced autonomic impairment in the DM group, the direction of these findings may appear counterintuitive and suggests that factors beyond sympathovagal imbalance contribute to BP variability in this cohort. BP variability is influenced not only by autonomic regulation but also by vascular structure and stiffness, atherosclerosis burden, and antihypertensive therapy [51]. Notably, as shown in Table 2, patients in the DM group had a significantly higher proportion of DHP-CCAs use. DHP-CCAs represent one of the most commonly prescribed antihypertensive drug classes and, alongside ACE inhibitors and angiotensin receptor blockers, are considered first-line therapy in patients with diabetes mellitus [52]. Moreover, in the study by Webb et al., DHP-CCAs were shown to have the strongest effect on reducing interindividual variability of systolic blood pressure, which may partly explain the lower values of ambulatory blood pressure variability parameters observed in the DM group [53]. Nevertheless, further dedicated studies are warranted to better elucidate the mechanisms underlying these findings, particularly in the context of the complex challenges related to optimal blood pressure regulation in patients with AS [20].

Even in a predominantly mechanical valvular condition such as severe AS, ECG-derived autonomic markers may provide clinically relevant information beyond conventional structural assessment. Reduced HRV and altered nonlinear indices may reflect underlying autonomic and electrophysiological vulnerability and were more pronounced in patients with DM. Prior studies have suggested that impaired pre-procedural HRV may be associated with less favorable outcomes after TAVR, supporting the potential prognostic relevance of autonomic ECG metrics in this setting [54]. Collectively, these observations indicate that advanced electrocardiographic phenotyping could contribute to refined risk stratification in severe AS and should be evaluated in prospective studies.

Given the exploratory nature of this study and the relatively small sample size typical for HRV analyses in patients with severe AS undergoing TAVR, the results should be interpreted with caution. Multiple HRV parameters were analyzed to provide a comprehensive assessment of autonomic function; however, many of these measures reflect related aspects of the same physiological signal and are not fully independent. Therefore, the findings should be considered hypothesis-generating and require confirmation in larger prospective studies.

### Study Limitations

Several limitations of the present study should be acknowledged. First, the relatively limited sample size may have reduced the statistical power to detect more subtle differences between groups. Future studies with larger patient cohorts are warranted to confirm these findings, ideally using a longitudinal design to assess their prognostic implications over time. Another limitation of the relatively small sample size is the large number of HRV, nonlinear, ABPM, and ECG parameters analyzed. Although these variables were included to provide a comprehensive assessment of autonomic function, many of them reflect related aspects of the same physiological signal and are therefore not fully independent. Consequently, the possibility of type I error cannot be completely excluded, particularly for results with borderline *p*-values, and the findings should be interpreted as exploratory and hypothesis-generating. Second, DM was treated as a binary variable in the present analysis. Additional diabetes-related characteristics, such as glycated hemoglobin (HbA1c), duration of diabetes, presence of microvascular complications and detailed antidiabetic therapy, were not systematically analyzed and should be considered in future studies. Third, although ANS function was evaluated using short- and long-term ECG-derived parameters and ambulatory blood pressure monitoring, not all autonomic testing modalities could be applied. Head-up tilt testing and standardized cardiovascular reflex tests are limited or contraindicated in patients with AS, particularly in those with severe disease. Future studies may incorporate alternative methods, such as baroreceptor sensitivity assessment or impedance cardiography, to provide additional insight into autonomic regulation and hemodynamic characteristics in this patient population.

## 5. Conclusions

Autonomic nervous system function is altered in patients with severe aortic stenosis and appears to be more profoundly impaired in those with concomitant diabetes mellitus, a condition strongly associated with cardiac autonomic neuropathy. Compared with non-diabetic patients, individuals with diabetes exhibited more pronounced reductions in both linear and nonlinear autonomic parameters, predominantly reflecting diminished parasympathetic modulation, as assessed by short- and long-term ECG-derived indices. Differences in ambulatory blood pressure variability further suggest that blood pressure regulation may differ between diabetic and non-diabetic patients with severe aortic stenosis, although such variability is influenced by multiple mechanisms beyond autonomic control.

Overall, these findings indicate that diabetes mellitus is associated with accentuated autonomic dysfunction in severe aortic stenosis and support the potential value of advanced ECG-derived autonomic metrics as complementary tools for pathophysiological characterization beyond conventional clinical and echocardiographic assessment. However, given the exploratory design of the present study and the relatively limited sample size typical for HRV analyses in this population, these findings should be considered hypothesis-generating and warrant confirmation in larger prospective studies.

## Figures and Tables

**Table 1:** Demographic and clinical characteristics of the study population.

	DM *N* = 21	Non DM *N* = 53	*p* Value
Female (*n*, %)	12 (57.1%)	28 (52.8%)	0.737 ^c^
Age (mean ± SD)	77 ± 5.1	78.2 ± 7.4	0.473 ^t^
BMI (kg/m^2^)	28.5 ± 5.1	25.5 ± 4.2	0.010 ^t^
Hypertension (*n*, %)	21 (100%)	52 (98.1%)	1.000 ^f^
HLP (*n*, %)	20 (95.2%)	49 (79.2%)	0.160 ^f^
Ex and active smokers (*n*, %)	9 (42.9%)	14 (26.3%)	0.168 ^c^
CAD (*n*, %)	12 (57.1%)	27 (50.9%)	0.630 ^c^
Previous MI (*n*, %)	1 (4.8%)	0	0.284 ^f^
Previous PCI (*n*, %)	2 (9.5%)	9 (17%)	0.718 ^f^
Previous ACB (*n*, %)	3 (14.3%)	7 (13.2%)	1.000 ^f^
Carotid artery disease (*n*, %)	8 (38.1%)	11 (20.8%)	0.124 ^c^
Paroxsysmal AF (*n*, %)	1 (4.8%)	8 (15.1%)	0.431 ^f^
NYHA I (*n*, %)	2 (9.5%)	3 (5.7%)	0.587 ^f^
NYHA II (*n*, %)	14 (66.7%)	32 (60.4%)	
NYHA III (*n*, %)	5 (23.8%)	18 (34%)	
Angina (*n*, %)	7 (33.3%)	24 (45.3%)	0.348 ^c^
Previous Syncope (*n*, %)	0	6 (11.3%)	0.175 ^f^
AV block (*n*, %)	4 (19%)	11 (20.8%)	1.000 ^f^
LBBB (*n*, %)	0	1 (1.9%)	0.683 ^f^
RBBB (*n*, %)	3 (14.3%)	4 (7.5%)	
IVCD (*n*, %)	2 (9.5%)	9 (17%)	

DM—Diabetes Mellitus; BMI—Body mass index; kg—kilograms; m^2^—meter squared; HLP—Hyperlypoproteinemia; CAD—Coronary Artery Disease; MI—Myocardial Infarction; PCI—Percutanenous Coronary Intervention; ACB—Arterycoronary bypass; AF—Atrial Fibrilation; NYHA—New York Heart Association; SD—Standard deviation; ^c^—Pearson Chi-Square; ^t^—Independent Student’s *t*-test; ^f^—Fisher’s Exact test.

**Table 2:** Medications used in the study population.

	DM *N* = 21	Non DM *N* = 53	*p* Value
ACE inhibitors (*n*, %)	12 (57.1%)	37 (71.2%)	0.249 ^c^
ARB (*n*, %)	4 (19%)	4 (7.7%)	0.216 ^c^
ARNI (*n*, %)	2 (9.5%)	2 (3.8%)	0.574 ^f^
BB (*n*, %)	19 (90.5%)	44 (84.6%)	0.714 ^f^
DHP-CCAs (*n*, %)	13 (61.9%)	18 (34.6%)	0.033 ^c^
MRA (*n*, %)	7 (33.3%)	9 (17.3%)	0.209 ^f^
SGLT 2 inhibitors (*n*, %)	6 (28.6%)	17 (32.7%)	0.732 ^f^
Loop diuretics (*n*, %)	8 (38.1%)	26 (50%)	0.356 ^c^
Thiazide diuretics (*n*, %)	4 (19%)	12 (23.1%)	1.000 ^f^
Long-acting nitrates (*n*, %)	2 (9.5%)	1 (1.9%)	0.197 ^f^
Trimetazidine (*n*, %)	3 (14.3%)	9 (17.3%)	1.000 ^f^
ASA (*n*, %)	15 (71.4%)	37 (71.2%)	0.981 ^c^
Clopidogrel (*n*, %)	4 (19%)	8 (15.4%)	0.734 ^f^

DM—Diabetes Mellitus; ACE—Angiotensin-Converting Enzyme; ARB—Angiotensin II Receptor Blocker; ARNI—Angiotensin Receptor–Neprilysin Inhibitor; BB—Beta Blockators; DHP-CCA—Dihydropyridine Calcium Channel inhibitors; MRA—Mineralocorticoid Receptor Antagonist; SGLT2—Sodium Glucose Co-Transporter 2; ASA—Acetylsalicylic Acid; ^c^—Pearson Chi-Square; ^f^—Fisher’s Exact test.

**Table 3:** Echocardiographic parameters of the study population.

	DM *N* = 21	Non-DM *N* = 53	*p* Value
LV EF (%) (mean ± SD)	51.2 ± 8.6	50.1 ± 9.6	0.652 ^t^
LV EDD (mm) (mean ± SD)	50.8 ± 5.5	51.1 ± 5.5	0.317 ^t^
LV ESD (mm) (mean ± SD)	32.4 ± 5.9	33.3 ± 6.2	0.577 ^t^
LA (mm) (mean ± SD)	41.1 ± 5.1	39.7 ± 4.8	0.306 ^t^
Mean PG (mmHg)	56.7 ± 13.3	52.4 ± 12.7	0.201 ^t^
Max PG (mmHg)	91.1 ± 19.9	86 ± 19.3	0.310 ^t^
AVA VTI (mean ± SD)	0.7 ± 0.2	0.7 ± 0.2	0.346 ^t^
IVS (mm) (mean ± SD)	11.6 ± 1.7	11.3 ± 1.5	0.625 ^t^
Asc. Ao (mm) (mean ± SD)	34.8 ± 5.4	33.9 ± 4.3	0.445 ^t^
RVSP (mmHg) (mean ± SD)	38.8 ± 13.1	38.8 ± 10.1	0.994 ^t^
WMA of basal IVS (*n*, %)	2 (9.5%)	7 (13.7%)	0.343 ^f^
WMA of others part of IVS (*n*, %)	1 (4.8%)	1 (2%)	
WMA of whole IVS (*n*, %)	2 (9.5%)	1 (2%)	
Other WMA (*n*, %)	9 (42.9%)	16 (31.4%)	0.352 ^c^

DM—Diabetes Mellitus; LV—Left Ventricle; EF—Ejection fraction; EDD—End diastolic diameter; ESD—End systolic diameter; LA—Left atria; PG—Transaortic pressure gradient; AVA—Aortic Valve Area; VTI—Velocity Time Integral; IVS—Interventricular Septum; Asc. Ao—Ascending Aorta; RVSP—Right Ventricle Systolic Pressure; WMA—Wall motion abnormalities; ^t^—Independent Student’s *t*-test; ^f^—Fisher’s Exact test; ^c^—Pearson Chi-Square.

**Table 4:** ECG and short-term Heart Rate Variability parameters of the study population.

	DM *N* = 21	Non-DM *N* = 53	*p* Value
HR (bpm) (mean ± SD)	69.2 ± 11.6	67.4 ± 13.3	0.583 ^t^
P wave (ms) (mean ± SD)	94.8 ± 18	107.8 ± 20	0.012 ^t^
PQ (ms) (Mdn (IQR))	162.7 ± 29.5	174.2 ± 41.1	0.251 ^t^
QRS (ms) (Mdn (IQR))	87 (81.5–107.5)	89 (84–101)	0.598 ^m^
T wave (ms) (mean ± SD)	284 ± 39.4	299.8 ± 33.3	0.087 ^t^
QTc (ms) (mean ± SD)	434 ± 33.4	445.6 ± 38.9	0.279 ^t^
SDNN (ms) (Mdn (IQR))	57 (20.5–107)	71 (42.5–158.5)	0.143 ^m^
RMSSD (ms) (Mdn (IQR))	67 (16.5–148)	98 (48–227)	0.132 ^m^
PNN50 (ms) (Mdn (IQR))	3 (0–11)	8 (1.5–21.5)	0.058 ^m^
TI (Mdn (IQR))	5.9 (4.4–8.1)	6.5 (4.3–8.7)	0.533 ^m^
TP (ms^2^) (Mdn (IQR))	483.7 (189.8–1366.9)	667.6 (356.3–1285.5)	0.301 ^m^
VLF (ms^2^) (Mdn (IQR))	270.6 (86.8–598)	226.6 (129–501.2)	0.607 ^m^
LF (ms^2^) (Mdn (IQR))	88.2 (37.2–258.4)	172.5 (63.7–300.6)	0.123 ^m^
HF (ms^2^) (Mdn (IQR))	118 (22.9–194.1)	154.1 (71.6–314.4)	0.145 ^m^
LF/HF (Mdn (IQR))	1.2 (0.5–2.1)	0.8 (0.5–1.6)	0.687 ^m^

DM—Diabetes Mellitus; HR—Heart rate; bpm.—beats per minute; ms—milliseconds; SDNN—Standard deviation of normal RR intervals; RMSSD—Root Mean Square of Successive Differences; PNN50—Percentage of successive NN intervals that differ by more than 50 milliseconds; TI—Triangular index; TP—Total power; ms^2^—milliseconds squared; VLF—Very Low Frequency; LF—Low Frequency; HF—High frequency; SD—Standard deviation; Mdn—Median; IQR—Interquartile range (25–75%); ^t^—Independent Student’s *t*-test; ^m^—Mann–Whitney U test.

**Table 5:** 24H Holter ECG and long-term Heart Rate Variability parameters of the study population.

	DM *N* = 21	Non-DM *N* = 53	*p* Value
HR (bpm) (mean ± SD)	67.1 ± 8.6	63.1 ± 9.9	0.107 ^t^
SDNN (ms) (mean ± SD)	104.7 ± 42.7	126.9 ± 32.8	0.041 ^t^
SDANN (ms) (mean ± SD)	84.3 ± 33.1	102.9 ± 31	0.026 ^t^
SDNN Ind. (ms) (mean ± SD)	51.2 ± 34.2	60.4 ± 24.3	0.205 ^t^
RMSSD (ms) (Mdn (IQR))	31 (19–55)	52 (28.5–88)	0.061 ^m^
PNN50 (ms) (Mdn (IQR))	3 (0–10.5)	9 (3.5–19)	0.014 ^m^
TI (mean ± SD)	24.2 ± 10.7	30.4 ± 9.3	0.015 ^t^
TP (ms^2^) (Mdn (IQR))	1609 (1274.1–3026.6)	1751.5 (1274.1–3026.6)	0.081 ^m^
ULF (ms^2^) (Mdn (IQR))	12.4 (6.7–35.4)	21.6 (12–57.1)	0.043 ^m^
VLF (ms^2^) (Mdn (IQR))	1054.4 (476.2–1664.6)	1333.5 (824–2200)	0.104 ^m^
LF (ms^2^) (Mdn (IQR))	189 (71–464.8)	273 (158.2–473.9)	0.090 ^m^
HF (ms^2^) (Mdn (IQR))	84.5 (42.7–226.5)	137.1 (80–350.1)	0.049 ^m^
LF/HF (Mdn (IQR))	2.1 (1.5–2.9)	2.1 (1.4–2.9)	0.502 ^m^
PVC (Mdn (IQR))	31 (0.5–143.5)	25 (2–311.5)	0.665 ^m^
PAC (Mdn (IQR))	74 (29–303)	152 (57.6–881)	0.195 ^m^
Abnormal TO PVC * (*n*,%)	6 (50%)	8 (25.8%)	0.160 ^f^
Abnormal TS PVC (*n*,%)	6 (50%)	9 (29%)	0.287 ^f^
Both abnormal TO and TS (*n*,%)	4 (33.3%)	3 (9.7%)	0.081 ^f^
DC (ms^2^) (Mdn (IQR))	3.9 (3.4–5.9)	4.4 (3.4–5.8)	0.610 ^m^
AC (ms^2^) (Mdn (IQR))	4.6 (3.8–8.3)	6.2 (5.2–7.5)	0.079 ^m^

DM—Diabetes Mellitus; HR—Heart rate; bpm.—beats per minute; ms—milliseconds; SDNN—Standard deviation of normal RR intervals; RMSSD—Root Mean Square of Successive Differences; PNN50—Percentage of successive NN intervals that differ by more than 50 milliseconds; TI—Triangular index; TP—Total power; ms^2^—milliseconds squared; ULF—Ultra Low Frequency; VLF—Very Low Frequency; LF—Low Frequency; HF—High frequency; PVC—Premature Ventricular Contraction; PAC—Premature Atrial Contraction; TO—Turbulence Onset; TS—Turbulence Slope; DC—Deceleration Capacity; AC—Acceleration Capacity; *—For TO and TS, percentage refers to the number of patients in whom the parameter was calculated (DM = 12, non-DM = 31); SD—Standard deviation; Mdn—Median; IQR—Interquartile range (25–75%); ^t^—Independent Student’s *t*-test; ^m^—Mann–Whitney U test; ^f^—Fisher’s Exact test.

**Table 6:** Poincare plot analysis parameters of the study population.

	DM *N* = 21	Non DM *N* = 53	*p* Value
VLI (ms) (Mdn (IQR))	126.9 (100.2–186.1)	165.4 (130.8–199.4)	0.027 ^m^
VAI (degree) (Mdn (IQR))	0.2 (0.1–0.4)	0.3 (0.2–0.8)	0.033 ^m^
L (ms) (Mdn (IQR))	562 (386–679.5)	648 (554.5–761)	0.007 ^m^
D (ms) (Mdn (IQR))	125 (58–183.5)	171 (93–226)	0.022 ^m^
SD1 (ms) (Mdn (IQR))	18 (12–34.5)	27 (18–50.5)	0.055 ^m^
SD2 (ms) (Mdn (IQR))	126 (100–185.5)	165 (130–198.5)	0.026 ^m^
SD1/SD2 (Mdn (IQR))	0.2 (0.1–0.3)	0.2 (0.1–0.3)	0.291 ^m^
SD2/SD1 (Mdn (IQR))	6.6 (3.6–10.4)	5.6 (3.3–8.5)	0.275 ^m^
LA (ms) (Mdn (IQR))	806 (606.5–960.5)	916 (762–1075.5)	0.017 ^m^
SA (ms) (Mdn (IQR))	171 (77.5–253.3)	242 (125–320)	0.022 ^m^

DM—Diabetes Mellitus; VLI—Vector Length Index; ms—milliseconds; VAI—Vector Angle Index; L—Poincaré Length; D—Poincare Dispersion; SD—Standard Deviation; LA—Poincaré Length; SA—Poincare width; Mdn—Median; IQR—Interquartile range (25–75%); ^m^—Mann–Whitney U test.

**Table 7:** Ambulatory blood pressure monitoring parameters of the study population.

	DM *N* = 21	Non-DM *N* = 53	*p* Value
**Average values**			
SBP (mean ± SD)	121.8 ± 13.3	124.5 ± 14.6	0.468 ^t^
SBPD (mean ± SD)	122.1 ± 13.4	124.8 ± 14.5	0.465 ^t^
SBPN (mean ± SD)	120.7 ± 14.6	123.2 ± 16.3	0.533 ^t^
DBP (mean ± SD)	62.3 ± 6.2	63.9 ± 7.5	0.397 ^t^
DBPD (Mdn (IQR))	61 (59–66)	66 (59–70)	0.275 ^m^
DBPN (mean ± SD)	58.9 ± 6.7	60.1 ± 7.2	0.307 ^t^
SD of SBPD (mean ± SD)	11.6 ± 4.1	14.5 ± 4.2	0.009 ^t^
SD of SBPN (Mdn (IQR))	8 (6–11.5)	11 (7–14)	0.090 ^m^
SD of DBPD (mean ± SD)	9.4 ± 4.3	11.6 ± 5	0.073 ^t^
SD of DBPN (mean ± SD)	6.5 ± 3.4	9.7 ± 5.7	0.018 ^t^
**Dipping status of SBP**			
Dipper (*n*, %)	1 (4.8%)	2 (3.9%)	0.905 ^c^
Non-dipper (*n*, %)	13 (61.9%)	29 (56.9%)	
Reverse-dipping (*n*, %)	7 (33.3%)	20 (39.2%)	
Dipping status of DBP			
Dipper (*n*, %)	4 (19%)	14 (27.5%)	0.750 ^f^
Non-dipper (*n*, %)	13 (61.9%)	29 (56.9%)	
Reverse-dipping (*n*, %)	3 (14.3%)	7 (13.7%)	
Extreme-dipping (*n*, %)	1 (4.8%)	1 (2%)	
% of pressure values above threshold *			
SBPD (*n*, %)	5 (23.8%)	25 (49%)	0.049 ^c^
SBPN (*n*, %)	12 (57.1%)	34 (66.7%)	0.444 ^c^
DBPD (*n*, %)	2 (9.5%)	5 (9.8%)	1.000 ^f^
DBPN (*n*, %)	7 (33.3%)	18 (35.3%)	0.874 ^c^

DM—Diabetes Mellitus; SBP—Systolic Blood pressure; SBPD—Systolic blood pressure during daytime; SBPN—Systolic blood pressure during night; DBP—Diastolic blood pressure; DBPD—Diastolic Blood Pressure during daytime; DBPN—Diastolic blood pressure during night; SD—Standard deviation; *—threshold is defined as 140 mmHg for SBP during day, or 125 mmHg during night for DBP, threshold for DBP if 90 mmHg during daytime, or 80 mmHg during night; Mdn—Median; IQR—Interquartile range (25–75%); ^t^—Independent Student’s *t*-test; ^m^—Mann–Whitney U test; ^c^—Pearson Chi Square; ^f^—Fisher Exacts test.

## Data Availability

The original contributions presented in this study are included in the article. Further inquiries can be directed to the corresponding author.
